# Physical Fitness and Body Composition in 10–12-Year-Old Danish Children in Relation to Leisure-Time Club-Based Sporting Activities

**DOI:** 10.1155/2018/9807569

**Published:** 2018-12-27

**Authors:** Christina Ørntoft, Malte Nejst Larsen, Mads Madsen, Lene Sandager, Ida Lundager, Andreas Møller, Lone Hansen, Esben E. Madsen, Anne-Marie Elbe, Laila Ottesen, Peter Krustrup

**Affiliations:** ^1^Department of Sports Science and Clinical Biomechanics, SDU Sport and Health Sciences Cluster (SHSC), University of Southern Denmark, Odense, Denmark; ^2^Team Danmark, Brøndby, Denmark; ^3^Department of Public Health, Sport Science, Aarhus University, Aarhus, Denmark; ^4^Department of Nutrition, Exercise and Sports, Copenhagen Centre of Team Sport and Health, University of Copenhagen, Copenhagen, Denmark; ^5^Department of Physiotherapy and Occupational Therapy, Faculty of Health and Technology, University College Copenhagen, Copenhagen, Denmark; ^6^Institute of Sport Psychology and Physical Education, Faculty of Sport Science, Leipzig University, Leipzig, Germany; ^7^Sport and Health Sciences, Faculty of Life and Environmental Sciences, University of Exeter, Exeter, UK

## Abstract

This study investigated whether the physical fitness and body composition of 10–12-year-old Danish children are related to participation in leisure-time club-based sporting activities. The study involved 544 Danish 10–12-year-old 5th-grade municipal schoolchildren (269 boys and 275 girls, 11.1 ± 0.4 years). After answering a questionnaire about leisure-time sporting activities, the children were divided into four groups: football club participation (FC; n=141), other ball games (OBG; n=42), other sports (OS; n=194), and no sports-club participation (NSC; n=167). The children completed a battery of health and fitness tests, including a 20 m sprint test, a standing long-jump test, the Yo-Yo IR1 children's test (YYIR1C), and body composition, blood pressure, resting heart rate (HR_rest_), and the flamingo balance test. The children engaged in club-based ball games (FC and OBG) had higher (p<0.05) lean body mass than NSC (FC: 17.5 ± 2.9; OBG: 18.4 ± 2.6; OS: 16.7 ± 2.9; NSC: 16.4 ± 2.8 kg), performed better (p<0.05) in the YYIR1C test (FC: 1083 ± 527; OBG: 968 ± 448; OS: 776 ± 398; NSC: 687 ± 378 m), and had lower (p<0.05) %HR_max_ after 1, 2, and 3 min of YYIR1C. Moreover, HR_rest_ was lower (p<0.05) for FC than for OS and NSC (FC: 68 ± 9 vs OS: 72 ± 10 and NSC: 75 ± 10 bpm), and lower (p<0.05) for OBG than for NSC (OBG: 70 ± 10 vs NSC: 75 ± 10 bpm). This study found that 10–12-year-old Danish children engaged in club-based football and other ball games had better exercise capacity, lower resting heart rate, and higher muscle mass than children not engaged in leisure-time sports. Thus, participation in club-based leisure-time ball-game activities seems to be of importance for the fitness and health profile of prepubertal children.

## 1. Introduction

It is known that precursors of adult cardiovascular disease (CVD) begin in childhood and that paediatric obesity, an important influence on overall CVD risk, tracks to later life and is associated with decreased quality of life and early mortality [1, 2]. There is an association between clustering of CVD risk factors and physical inactivity as well as poor fitness in 9–16-year-old schoolchildren that several studies emphasise [3, 4]. Physical activity plays an important role in the prevention of various chronic diseases that are challenging modern society [5] and it is evident that physical activity on a regular basis is associated with significant positive physical and mental health benefits [6].

Organised leisure-time sports-club participation is one potential way of increasing overall physical activity and fitness in young people, though other approaches can also be used, including physical education at school, school playground activities, and nonorganised leisure-time activities. Studies have shown that sports-club membership predicts higher levels of leisure-time physical activity [7, 8], and organised sport increases the probability of participants meeting the World Health Organization (WHO) global recommendation of 60 min of moderate-to-vigorous-intensity daily physical activity [7, 9]. It is emphasised by other studies that organised leisure-time sporting participation is associated with increased health-related physical activity and compliance with international physical activity guideline recommendations for children [10, 11]. In fact, as many as 98% of 9–11-year-old Danish children who participate in 1–4 weekly training sessions at local football clubs meet the recommendation on daily physical activity, with 83% for those participating in other sports-club activities and 76% for children not involved in sports-club activities [10].

There are some indications that physical fitness and body composition in children are influenced positively by sports-club participation, at least in the case of certain sports, compared to age-matched children not participating in organised leisure-time sports-club activities. Several training studies have shown that short-term (6–26 weeks) team-sport training improves intermittent exercise performance and aerobic fitness [12] and elicits significant adaptations of myocardial structure and function, in 8–12-year-old children [13]. Two Portuguese studies have shown that aerobic fitness evaluated by performance in the Yo-Yo intermittent recovery test is much higher in 9–16-year-old girls and boys participating in football-club training than in age-matched children not active in sports clubs [14, 15]. Specifically, it was observed that performance in the Yo-Yo intermittent recovery level 1 children's test (YYIR1C) was 43% and 57% higher in 8–10-year-old girls and boys, respectively, active in football clubs [14, 15]. These two studies also showed markedly lower fat percentages in the girls and boys active in football clubs in the age groups 12–13 and 14–16, whereas there were no significant differences in fat percentage in 9–11-year-olds. Another study carried out in Southern Europe has shown that 10–15-year-old children involved in football on a regular basis have stronger bones than children not participating in sports-club activities [16]. A recent investigation in younger (8–10-year-old) Danish schoolchildren has shown that children engaged in leisure-time sports-club activity, especially ball games, have better physical fitness and a healthier body composition than those not active in sports clubs. This was evidenced by superior aerobic and musculoskeletal fitness, sprint performance, and postural balance, as well as markedly lower fat mass index. Furthermore, the study showed that 8–10-year-old girls have better postural balance, though poorer aerobic fitness and sprint performance as well as lower bone mineralisation, than boys of the same age [17]. Since Hebert et al. [10] showed that participation in a football-club activity just once a week was positive in terms of living up to the recommendations for daily physical activity, while participation in other team sports was not, this study will separate football from other team sports. Also, it is interesting to investigate the status of 5th-graders compared to the younger children in the previous study, since physical activity levels are lowered from 3rd to 5th grade in terms of both organised and nonorganised physical activity [18].

The aim of the present study was therefore to investigate whether the physical fitness and body composition of 10–12-year-old Danish boys and girls was related to the type of voluntary sports-club activity, i.e., participation in football, other ball games, and other sports versus school-children with no sports-club involvement.

## 2. Methods

### 2.1. Experimental Approach to the Problem

The study used a cross-sectional design, including physical testing and basal information, to test whether physical fitness and body composition are associated with a specific type of sports-club participation, e.g., football, other ball games, other sports, and no sports-club participation.

### 2.2. Subjects

Five hundred and forty-four Danish 10–12-year-old 5th-grade schoolchildren (269 boys and 275 girls) participated in the study. The children were aged 11.1 ± 0.4 years, were 150.5 ± 7.2 cm tall, and weighed 41.5 ± 8.4 kg. They came from nine different schools, five located in the capital regions of Frederiksberg and Copenhagen municipalities and four in the countryside regions of Frederikssund and Roskilde municipalities, about 40 km away from the capital.

The study was approved by the Committees on Biomedical Research Ethics for the Capital Region of Denmark (J.no. H-15008117). Child assent and written informed parental consent were obtained for all participants. The children were tested during weeks 3–6 of the school year. All tests were performed by university staff members, supported by the schoolteachers.

### 2.3. Test Battery

All the testing was performed in the late summer (August to September) at the beginning of 5th grade. The children answered two questions about their leisure-time physical activity: Do you do any sports in your spare time as a member of a sports club? If so, which? The children were divided into four different groups according to their leisure-time sports-club participation: football club (FC); other ball games (OBG; e.g., handball, floorball, and basketball); other sports (OS; e.g., gymnastics, dancing, martial arts, tennis, badminton, volleyball, and riding); no sports-club participation (NSC). Ball games were defined as invasive ball games.  Table 1 shows the number of children participating in the four different categories.

In addition to the questionnaire, the children underwent measurements of body composition (height, weight, lean body mass, and percent body fat), resting blood pressure, and resting heart rate (HR_rest_) as well as performing several exercise tests (20 m sprint, YYIR1C, and horizontal jump). The tests were completed over two consecutive days with a requirement that no physical activity should be performed on the day before test day 1. Test day 1 included two 20 m sprints, two maximal horizontal jumps, and the YYIR1C. Before testing on test day 1, the children performed a standardised warm-up consisting of two sets of exercises 1 (forward running), 4 (circling partners), 12.1 (running 2 cones forward and 1 backwards), and 13 (high-speed running) from the FIFA 11+ warm-up programme [19]. Test day 2 involved measurements of resting heart rate, resting blood pressure, height and weight, and the flamingo balance test.

### 2.4. Test Day 1

#### 2.4.1. 20 m Sprint Test

After the warm-up, the children performed 2 x 20 m maximal sprints with at least 2 min of recovery between sprints. All sprints started from a standing position and were timed using two ports of light sensors (Witty Microgate, Bolzano, Italy) placed at 0 m (positioned 30 cm in front of the standing-start position) and at 20 m. The best time recorded was noted as the test result. The 20 m sprint has been shown to be a valid and reliable method for young children [20].

#### 2.4.2. Maximal Horizontal Jump Length

The warm-up included instructions about completing the squat jump from the akimbo position. The children stood upright with their toes just behind a line with their feet parallel and shoulder-width apart; after flexing the knees to the squat position and holding the position for at least 2 s, the children jumped as far as they could and the distance from the start line to the heel position was measured. Using a measuring tape, the jump length was measured to the nearest centimetre. Each child had two attempts. If they failed to perform two correct jumps, they were allowed an additional attempt. The longest jump was noted as the test result. The maximal horizontal jump length test is reliable and has been shown to be strongly associated with lower- and upper-body maximal strength [21, 22].

#### 2.4.3. Yo-Yo Intermittent Recovery Level 1 Children's Test (YYIR1C)

The YYIR1C test was performed indoors on one half of a wooden-floor handball court in accordance with the studies by Ahler, Bendiksen, Krustrup & Wedderkopp [23] and Bendiksen, Ahler, Clausen, Wedderkopp & Krustrup [24]. The test consisted of 2 × 16 m shuttle runs back and forth between cones placed 16 m apart (at the start/finish line and turning line) at progressively increasing speeds, interspersed by 10 s of jogging after each running bout around a cone placed 4 m behind the start/finish line. The total duration of the test varied from 2 to 22 min. The width of the running lanes was 1.3 m and each university staff member tested 9–12 pupils. The test was terminated when a pupil had failed twice to reach the finish line in time. The distance covered was recorded as the test performance. Before the test, the participants were thoroughly informed about the test protocol and tried out the first three running bouts of the test to get acquainted with the initial running speeds. HR was recorded throughout the test to determine relative HR loading after 1, 2, and 3 min. Submaximal HR (HR_submax_) was calculated as a mean over a 30 s period 15 s before to 15 s after 1, 2, and 3 min of exercise. HR_max_ was determined as the highest heart rate reached over a 15 s period during the YYIR1C test, as this test has been shown to be optimal for determining HR_max_ in 6-12-year-old children [14, 15, 24]. Submaximal aerobic loading and YYIR1C performance have both been shown to be valid measures of aerobic fitness [23–25]. The test–retest coefficient of variation of the YYIR1C has been shown to be 13% for 8–9-year-olds [23].

### 2.5. Test Day 2

#### 2.5.1. Resting Blood Pressure and Heart Rate

Arterial blood pressure was measured with the subjects in the supine position following at least 10 min of rest in a quiet room providing an optimal setting for relaxation. Blood pressure was recorded in mmHg as the average of three measurements on the left upper arm using an automatic blood pressure monitor (M6 HEM-7223-F, Omron, IL, USA), with the cuff size adjusted to the arm as appropriate. HR_rest_ was measured simultaneously in beats per minute(bpm) by a heart rate belt (POLAR Team2 system, Polar Electro Oy, Kempele, Finland). The lowest value obtained over a 15 s period was noted as the HR_rest_ value.

#### 2.5.2. Body Composition

Height was measured with 0.1 cm precision using a Tanita Leicester transportable stadiometer (Tanita, Amsterdam, Netherlands). Body mass, body fat percentage, and lean body mass were measured using an InBody 230 multifrequency body composition analyser (Biospace, California, USA). The InBody 230 has been validated in 7–12-year-old children, showing precise lean body mass but underestimated fat mass and fat percentage [26].

No maturation status assessment was performed. The subjects were weighed as described by Karelis, Chamberland, Aubertin-Leheudre, and Duval [27]. Data output, as calculated by the manufacturer's algorithm, included fat mass (kg), body fat (%), and lean body mass (kg).

#### 2.5.3. Flamingo Balance Test

Postural balance was assessed using a single-leg flamingo balance test [28]. The child was instructed to stand on one foot on a 50 cm-long, 3 cm-wide, and 5 cm-high metal bar with their eyes open, holding the contralateral leg at the ankle joint. The child was permitted to move their arms and nonstanding leg to assist balancing. The number of falls was counted and used as a measure of postural balance. A fall was defined as touching the ground, being unable to hold the contralateral leg at the ankle joint, or both. The child was given two attempts on each leg to choose their preferred leg before the number of falls was counted. The number of falls in 1 min was counted. If the child fell 20 times before the 1 min had expired, the time was noted and the number of falls in 1 min was calculated. The test has been proven to be valid and reliable as a test of balance in young children [29].

### 2.6. Statistical Procedures

Data are reported as means (±SD). All data were tested for normality. Between-group differences were tested using one-way analyses of variance (ANOVA) in Sigma Plot 13.0 (SYSTAT). When a significant interaction was detected, a Bonferroni post hoc test was used to locate the significant differences. Significance was accepted at p<0.05. Some of the children did not attend in one of the two test days or did not want to complete some of the tests (e.g., weight measures, YYIR1C), which means that the number of participants may differ between each test. When differences are presented in percentages, they are calculated as ((xx-NSC)/NSC).

## 3. Results

### 3.1. Resting Heart Rate and Blood Pressure

Children active in leisure-time club-based ball games (FC and OBG) had a 7 bpm lower (p<0.05) HR_rest_ than NSC children. FC also had a 4 bpm lower (p<0.05) HR_rest_ than OS (68 ± 9, 70±10, 72 ± 10, and 75 ± 10 bpm for FC, OBG, OS, and NSC, resp.; Table 2). FC girls had a 7 bpm lower (p<0.05) HR_rest_ than NSC girls, and FC boys had an 8 bpm lower (p<0.05) HR_rest_ than NSC boys (Table 3). There were no between-group differences in blood pressure (Tables 2 and 3).

### 3.2. Body Composition

Body composition variables related to height, weight, lean body mass, and fat percentage are presented in Table 2. Children active in club-based ball games (FC and OBG) had higher (p<0.05) lean body mass than NSC children (FC: 17.5 ± 2.9 kg and OBG: 18.4 ± 2.6 kg vs NSC: 16.4 ± 2.8 kg) but there were no significant between-group differences in BMI or body fat percentage (Table 2). OBG children were taller (p<0.05) than NSC children and heavier (p<0.05) than NSC and OS children. There were significant differences in body fat percentage between OS boys and NSC girls, with no other significant differences observed in relation to gender and leisure-time sports-club engagement (Table 3).

### 3.3. YYIR1C Test Performance

YYIR1C test performance was 57% and 41% better in FC and OBG than in NSC (p<0.05). FC performed 40% better than OS (1083 ± 527, 968 ± 448, 776 ± 398, and 687 ± 378 m for FC, OBG, OS, and NSC, resp.; Table 2). FC boys performed better (p<0.05) in the YYIR1C than OS and NSC boys.

After 1 min of the YYIR1C, all the three groups engaged in club-based sporting activities had lower (p<0.05) submaximal HR measured as %HR_max_ than NSC (Table 2). FC and OBG also had lower (p<0.05) submaximal values after 2 min and 3 min of the YYIR1C compared to NSC (Table 2). FC had a lower (p<0.05) submaximal heart rate after 3 min of the YYIR1C than OS. After 1 min of the YYIR1C, FC boys and OBG boys had lower (p<0.05) submaximal heart rate than NSC boys, whereas there were no between-group differences for the girls.

### 3.4. Sprint, Jump, and Balance Performance

Children engaged in club-based football had better (p<0.05) 20 m sprint performance (FC: 3.97 ± 0.25 s; OBG: 4.11 ± 0.26 s; OS: 4.05 ± 0.26 s; NSC: 4.13 ± 0.31 s) than the three other groups, with FC boys having better (p<0.05) performance than the NSC boys. FC girls had 9% better (p<0.05) horizontal jump performance than NSC girls. There were no other significant between-group differences for horizontal jump performance and postural balance (Table 2).

## 4. Discussion

The main findings of this study were that 10–12-year-old Danish children engaged in club-based leisure-time football and other ball games had better aerobic and muscular fitness than children not active in sports clubs. Furthermore, the children engaged in club-based football had better aerobic fitness than those engaged in other sports and were faster in 20 m sprinting compared to the other ball games. Most of these differences were present for girls as well as for boys, indicating that leisure-time ball-game activities are important for fitness and health profile in boys and girls aged 10–12 years. No significant differences were observed between groups, either overall or within gender, for fat percentage, BMI, horizontal jumping, and the flamingo balance test, except that those girls engaged in club-based football had higher horizontal jumping performance than girls in other sports or with no leisure-time sports.

The present study showed that children engaged in leisure-time football and other ball games had 9% and 7% lower HR_rest_ than NSC children. High HR_rest_ is associated with increased risk of cardiovascular morbidity [30–32]. An active lifestyle and participation in sports club in childhood tracks to adolescence and on into adulthood [32]. In the present study, HR_rest_ for children engaged in ball-game activities was similar to values observed in 8–10-year-old Danish schoolchildren, though HR_rest_ will decrease with increasing age [17, 33]. Moreover, the difference in HR_rest_ of 5-7 bpm between those involved in leisure-time ball-game activities and those with no sport club activity was also similar to the differences found between these groups for 8–10-year-olds [11], supporting the notion that ball-game activity is of importance for the aerobic fitness and health profile of children.

YYIR1C performance, which is known to be correlated to aerobic fitness in children [23, 24], was 63% (396 m) better in 10–12-year-old Danish children active in football clubs compared to those not engaged in leisure-time sports and 40% (307 m) better than those engaged in other sports. Based on the observed correlation between YYIR1C performance and directly measured maximal oxygen uptake in 8-9-year-olds (VO_2_max=0.116xYYIR1C+42.3 ml/min/kg; [23]), this difference can be roughly estimated to correspond to differences of 5 and 4 ml/min/kg in maximal oxygen uptake. The observed performance differences in the present study are greater than the differences observed in 9–11-year-old Portuguese girls (43%) and boys (57%) active in football clubs compared to untrained children [14, 15] and similar to the findings of a recent Danish study of 61% (350 m) better YYIR1C performance for 8–10-year-old Danish children active in ball games compared to children not active in sports clubs during their leisure-time [17]. In the present study, the children active in all sports-club activities had lower submaximal heart rate after 1 min of YYIR1C, while children participating in ball games also had lower submaximal heart rate after 2 min and 3 min. Overall, these findings, together with lower HR_rest_ and better YYIR1C performance, provide further support for the notion that participation in leisure-time football is an activity that 10-12-year-old children can perform to improve aerobic fitness level.

The differences in cardiovascular fitness could be related to the high amount of vigorous exercise carried out during ball games like football and handball [10, 11, 13, 34] and to the fact that children engaged in ball-game activities in their leisure time satisfy the physical activity recommendation of health authorities [35] in terms of both overall physical activity and time spent in moderate-to-vigorous activity [10, 11]. This is an extremely important finding considering the strong association between cardiovascular disease (CVD) risk scores and aerobic fitness measured, e.g., as VO_2peak_ during treadmill testing to exhaustion [36].

The present study also evaluated selected variables related to muscular fitness. The children playing football had 2–4% better 20 m sprint performance than the three other groups. One explanation for this could be the higher lean body mass, but the difference was only identified between FC and NSC, and those engaged in other team sports had poor sprinting performance despite high lean body mass values. Larsen et al. [17] observed 5% better sprint performance and better postural balance in the 8–10-year-old children active in ball games at sports clubs, accompanied by slightly lower body weight and significantly higher lean body mass. In the present study, we observed better horizontal jump performance for the girls participating in leisure-time football clubs compared to no leisure-time sports, but no other between-group differences. Further studies with more participants are warranted to evaluate performance in horizontal jump and flamingo balance test performance among subgroups, as the variability is larger in these tests compared to the sprint test.

The findings of higher lean mass for football players and no significant difference in fat mass are in accordance with the Portuguese studies by Povóas et al. [14, 15] for 9–11-year-old boys and girls engaged in football compared to those not active in sports clubs, whereas the footballers of both genders had higher lean mass and markedly lower fat percentages in the age groups 12–13 and 14–16. Moreover, the Portuguese study by Seabra et al. [16] showed that 10–15-year-old children involved in football-club activity on a regular basis had stronger bones than children not participating in leisure-time sport. This aspect is interesting given that the difference between children active in sports clubs and those not active in sports clubs may become greater in puberty as well as with an increasing number of years of sports-club activity. Pilgaard [18] studied Danish schoolchildren's participation in sports and found that adolescents are becoming less involved in both school sport and leisure-time sport compared to 2007, with a drop in participation from the age of 9 years to the age of 11 years. Furthermore, the analysis reveals a pattern of more individualised, self-organised, and flexible sports participation among adolescents. The current study, however, shows the importance of team sports for this age group and their physical health, adding support for the importance of sports clubs and other organisations in the health and fitness of prepubertal children and efforts to find a way to decrease the drop-out rate of adolescents.

A limitation of this study is the lack of information on the children's everyday activities (e.g., active transport and activities at school). Obviously, it is also worth noting that the sports-club participation is voluntary and that the children may have chosen to take part in the sports-club activity based on skills, interests, and prior exposure to sports. The number of participants in the present study is high for FG, OS, and NSC, ranging from 141 to 197 participants, but not as high for the other ball games, with 42 participants, giving a stronger conclusion on sports-club football engagement compared to the other ball games. A further limitation is that there is no evaluation of the link between the number of years the children have been active in sports clubs and their fitness and health profile. Finally, it should be considered that the body composition analyser chosen for the study is well correlated with DXA scan method but is shown to underestimate fat mass and body fat percentage [26]. This must be considered if the studied groups are compared with other groups who has used another device for measuring body composition.

In conclusion, this study showed that 10–12-year-old children engaged in club-based ball games during leisure time had lower resting heart rate, better exercise capacity, and higher lean body mass than children not engaged in leisure-time sports. Thus, participation in club-based leisure-time ball-game activities seems to be of importance for the fitness and health profile of prepubertal children.

## Figures and Tables

**Table 1:** Number and percentage of children in relation to participation in club-based leisure-time sporting activity.

	***Football***	***Other Ball Games***	***Other Sports***	***No Sports Club***	***Total***
***Boys***	97 (36.0%)	16 (6.0%)	75 (27.9%)	81 (30.1%)	269
***Girls***	44 (16.0%)	26 (9.4%)	119 (43.3%)	86 (31.3%)	275
***Total***	141 (25.9%)	42 (7.8%)	194 (35.6%)	167 (30.7%)	544

**Table 2:** Overall means ± standard deviations for body composition and fitness variables by participation groups.

	***Football*** ***(n = 141)***	***Other Ball games*** ***(n = 42)***	***Other Sports*** ***(n = 194)***	***No Sports Club*** ***(n = 167)***
***Height (cm)***	151.2 ± 7.2	153.6 ± 6.6^*∗*^	150.1 ± 7.4	149.5 ± 6.7

***Weight (kg)***	42.1 ± 8.1	44.7 ± 7.0^*∗*#^	40.6 ± 8.2	41.2 ± 9.1

***BMI***	18.3 ± 2.6	18.9 ± 2.3	17.9 ± 2.5	18.3 ± 3.2

***Lean mass (kg)***	17.5 ± 2.9^*∗*^	18.4 ± 2.6^*∗*#^	16.7 ± 2.9	16.4 ± 2.8

***Body fat percentage***	20.7 ± 7.2	21.9 ± 7.6	21.1 ± 6.8	23.1 ± 8.2

***YYIR1C (m)***	1083 ± 527^*∗*#^ (b = 80; g = 36)	968 ± 448^*∗*^ (b = 16; g = 26)	776 ± 398 (b = 54; g = 82)	687 ± 378 (b = 52; g = 60)

**H** **R**_**max**_ ***(bpm)***	203 ± 9 (b = 80; g = 36)	206 ± 9 (b = 16; g = 26)	204 ± 9 (b = 54; g = 82)	204 ± 9 (b = 52; g = 60)

***1 min submax*** %**HR**_**max**_	82 ± 5^*∗*^ (b = 80; g = 36)	81 ± 6^*∗*^ (b = 16; g = 26)	84 ± 6^*∗*^ (b = 54; g = 82)	86 ± 8 (b = 52 g = 60)

***2 min submax*** %**HR**_**max**_	89 ± 4^*∗*^ (b = 79; g = 36)	89 ± 4^*∗*^ (b = 16; g = 26)	91 ± 4 (b = 54; g = 82)	92 ± 6 (b = 49, g = 57)

***3 min submax*** %**HR**_**max**_	91 ± 4^*∗*#^ (b = 78, g = 35)	92 ± 4^*∗*^ (b = 14 and g = 18)	93 ± 3 (b = 49, g = 75)	95 ± 3 (b = 46, g = 53)

***20-m sprint (s)***	3.97 ± 0.25^*∗*#*α*^ (b = 93; g = 42)	4.11 ± 0.26 (b = 16; g = 26)	4.05 ± 0.26 (b = 70; g = 115)	4.13 ± 0.31 (b = 77; g = 82)

***Horizontal jump (cm)***	121.9 ± 15.5 (b = 93; g = 42)	120.8 ± 14.7 (b = 16; g = 26)	118.9 ± 17.4 (b = 70; g = 115)	114.0 ± 17.9 (b = 77; g = 82)

***Flamingo balance (falls/60 s)***	17.4 ± 9.2 (b = 93; g = 42)	17.8 ± 9.1 (b = 16; g = 26)	18.3 ± 10.3 (b = 70; g = 115)	19.6 ± 8.5 (b = 77; g = 82)

**H** **R**_**rest**_ *** (bpm)***	68 ± 9^*∗*#^ (b = 92; g = 42)	70 ± 10^*∗*^ (b = 15; g = 25)	72 ± 10 (b = 70; g = 115)	75 ± 10 (b = 74; g = 82)

***SBP (mmHg)***	108 ± 10 (b = 92; g = 42)	109 ± 10 (b = 15; g = 25)	106 ± 11 (b = 70; g = 115)	107 ± 11 (b = 74; g = 82)

***DBP (mmHg)***	63 ± 6 (b = 92; g = 42)	63 ± 6 (b = 15; g = 25)	63 ± 6 (b = 70; g = 115)	64 ± 7 (b = 74; g = 82)

***MAP (mmHg)***	78 ± 7 (b = 92; g = 42)	78 ± 7 (b = 15; g = 25)	77 ± 7 (b = 70; g = 115)	78 ± 7 (b = 74; g = 82)

b: boys; g: girls.

^*∗*^Significantly different from no sports club involvement

^#^Significantly different from other sports

^*α*^Significantly different from other ball games.

**Table 3:** Means ± standard deviations for body composition variables and physical performance by participation groups and gender.

	***Football***		***Other Ball Games***		***Other Sports***		***No Sports Club***	
	**Boys** **(n = 78-97)**	**Girls** ** (n = 35-44)**	**Boys** ** (n = 14-16)**	**Girls** ** (n = 18-26)**	**Boys** ** (n = 49-75)**	**Girls** **(n = 75-119)**	**Boys** ** (n = 49-81)**	**Girls** **(n = 53-86)**
***Height (cm)***	150.9 ± 7.1	151.0 ± 7.7	154.2 ± 6.7	152.3 ± 6.8	151.4 ± 7.4	149.5 ± 7.5	149.5 ± 6.2	149.4 ± 7.3

***Weight (kg)***	41.9 ± 8.9	42.2 ± 7.5	42.3 ± 6.0	44.3 ± 7.8	40.9 ± 7.8	40.5 ± 8.5	41.4 ± 9.0	40.9 ± 9.2

***BMI***	18.4 ± 2.9	18.2 ± 2.3	17.7 ± 1.9	18.9 ± 2.5	17.8 ± 2.4	17.9 ± 2.6	18.4 ± 3.3	18.2 ± 3.2

***Lean mass (kg)***	17.5 ± 2.8	17.4 ± 3.3	18.7 ± 2.7	18.1 ± 2.7	17.2 ± 2.9	16.4 ± 2.9	16.6 ± 2.4	16.1 ± 3.1

***Body fat***%	20.0 ± 8.1	21.9 ± 6.3	17.5 ± 5.8	24.1 ± 7.6	19.5 ± 6.5^¤^	22.0 ± 6.9	22.5 ± 8.1	23.6 ± 8.4

***YYIR1C (m)***	1178 ± 570	873 ± 386	1074 ± 473	904 ± 419	846 ± 421^£^	731 ± 377^£^	759 ± 434^£^	621 ± 295^*£*€^

***HRmax (bpm)***	203 ± 9	205 ± 9	204 ± 11	206 ± 8	202 ± 9	205 ± 8	202 ± 8	207 ± 9^$^

***1 min submax*** %**HR**_**max**_	82 ± 6	83 ± 5	80 ± 5	82 ± 7	84 ± 5	84 ± 6	87 ± 9^*£*€^	86 ± 6^*£*€^

***2 min submax*** %**HR**_**max**_	89 ± 6	90 ± 4	88 ± 4	90 ± 4	90 ± 4	91 ± 4	91 ± 8	92 ± 4^*£*€^

***3 min submax*** %**HR**_**max**_	91 ± 4	93 ± 3	91 ± 4	93 ± 4	93 ± 3	94 ± 3	94 ± 4	95 ± 3^*£*€^

***20-m sprint (s)***	3.97 ± 0.25	3.99 ± 0.27	4.03 ± 0.26	4.16 ± 0.26^£^	4.04 ± 0.28	4.06 ± 0.25	4.15 ± 0.34^£^	4.11 ± 0.29^£^

***Horizontal jump (cm)***	121.6 ± 14.7	122.5 ± 17.5	127.1 ± 13.3	115.9 ± 13.3	123.8 ± 18.1	115.0 ± 16.3^Δ^	116.2 ± 18.1	112.0 ± 17.8^*£*&€Δ^

***Flamingo test (falls/60 s)***	18.6 ± 9.6	14.8 ± 7.9	17.7 ± 7.2	18.0 ± 10.0	19.1 ± 10.5	17.8 ± 16.3	20.4 ± 7.6	18.8 ± 9.2

***Resting HR (bpm)***	67 ± 9	68 ± 9	69 ± 10	70 ± 10	70 ± 10	73 ± 10	74 ± 8^£^	76 ± 11^£&Δ^

***SBP (mmHg)***	108 ± 11	106 ± 8	108 ± 11	110 ± 10	108 ± 12	105 ± 11	109 ± 11	106 ± 11

***DBP (mmHg)***	63 ± 7	63 ± 6	63 ± 7	63 ± 6	62 ± 7	63 ± 6	64 ± 6	64 ± 7

***MAP (mmHg)***	78 ± 7	77 ± 6	78 ± 7	79 ± 7	77 ± 8	77 ± 7	79 ± 7	78 ± 7

^¤^Significantly different from NSC girls

^£^Significantly different from boys engaged in football clubs

^€^Significantly different from boys engaged in other ball games

^$^Significantly different from boys with no sports-club involvement

^&^Significantly different from girls engaged in football clubs

^Δ^Significantly different from boys playing other sports.

## Data Availability

The data used to support the findings of this study are available from the corresponding author upon request.

## Additional Points

*Practical Application. *The present results of this study suggest that club-based leisure-time ball-game activity involving football handball, basketball, and floorball have a positive impact on children's fitness and health profile, which, hopefully, will track into adulthood. These results should be taken into consideration by parents, sports organisations and politicians so that children's participation in sports clubs can be increased and the subsequent drop-out rate decreased. Furthermore, emphasis should be placed on reducing barriers for sports-club membership, e.g., parents' socioeconomic status, as this is a main factor especially for girls [37].
